# Practice and Preparation Time Facilitate System-Switching in Perceptual Categorization

**DOI:** 10.3389/fpsyg.2017.01964

**Published:** 2017-11-07

**Authors:** Sébastien Hélie

**Affiliations:** Department of Psychological Sciences, Purdue University, West Lafayette, IN, United States

**Keywords:** system-switching, preparation time, practice, perceptual categorization, task-switching

## Abstract

Mounting evidence suggests that category learning is achieved using different psychological and biological systems. While existing multiple-system theories and models of categorization may disagree about the number or nature of the different systems, all assume that people can switch between systems seamlessly. However, little empirical data has been collected to test this assumption, and recent available data suggest that system-switching is difficult. The main goal of this article is to identify factors influencing the proportion of participants who successfully learn to switch between procedural and declarative systems on a trial-by-trial basis. Specifically, we tested the effects of preparation time and practice, two factors that have been useful in task-switching, in a system-switching experiment. The results suggest that practice and preparation time can be beneficial to system-switching (as calculated by a higher proportion of switchers and lower switch costs), especially when they are jointly present. However, this improved system-switching comes at the cost of a larger button-switch interference when changing the location of the response buttons. The article concludes with a discussion of the implications of these findings for empirical research on system-switching and theoretical work on multiple-systems of category learning.

## 1. Introduction

Categorization is a ubiquitous process in daily life. From categorizing objects as edible or not to categorizing people as friends or enemies, everyday life is filled with thousands of category decisions. Over the past 20 years, mounting evidence has been gathered that category learning is achieved using a number of different psychological and biological systems (e.g., Nosofsky et al., 1994; Ashby et al., 1998; Erickson and Kruschke, 1998; Hélie et al., 2010; Waldschmidt and Ashby, 2011; Ashby and Valentin, 2017). While existing multiple-systems theories and models of categorization sometimes disagree about the number or nature of the different systems, all assume that people can switch between systems seamlessly. However, little empirical data has been collected to test this assumption.

### 1.1. System-switching in categorization

It took over a decade after the initial proposals of multiple-systems theories of categorization (Nosofsky et al., 1994; Ashby et al., 1998; Erickson and Kruschke, 1998) for the first empirical investigation of system-switching to be performed (Erickson, 2008). Erickson asked participants to categorize “space shuttle” schematics (i.e., rectangles with internal line segments) into one of four categories. Two of the categories could be distinguished using a simple verbal rule [i.e., rule-based (RB) categories] while the other two categories could not [i.e., information-integration (II) categories]. Each category was associated with a different response button, and categories (RB or II) were cued using the background color. Decision-bound models (Ashby, 1992; Maddox and Ashby, 1993; Hélie et al., 2017) were individually fit to the RB and II data to identify “switchers” and “non-switchers.” Switchers were participants whose RB and II data were best fit by optimal models (i.e., a decision bound on one of the stimulus dimensions for RB trials and the general linear classifier for the II trials). All other participants were labeled as non-switchers. Perhaps surprisingly, only 37% of the participants were able to switch categorization system on a trial-by-trial basis (Erickson, 2008).

Following these intriguing results, Ashby and Crossley (2010) tried to reproduce Erickson's (2008) results with three minor modifications: (1) stimuli were disks with sine-wave gratings instead of rectangles; (2) the categories were not cued by background color; and (3) only two response buttons were used. Specifically, participants were asked to categorize stimuli in one of two categories (each associated with a different response button), but depending on where the stimulus laid in the stimulus space (i.e., what the stimulus looked like), correct categorization required applying a declarative (for RB categories) or procedural (for II categories) strategy (as in Erickson, 2008). Not surprisingly, removing the separate response buttons and background color cues made the task much more difficult, and only 4% of the participants were identified as switchers using the same decision-bound modeling method as Erickson (2008).

More recently, Crossley et al. (2017) published a new experiment again using disks with sine-wave gratings but this time adding back the background color cue and separate response buttons (4 categories) used in Erickson (2008). Crossley et al. used more training trials, and obtained a proportion of switchers close to 40%, which is higher than Ashby and Crossley (2010) and close to Erickson (2008). Another contribution of the Crossley et al. study was that it made a connection between system-switching and task-switching (Kiesel et al., 2010; Vandierendonck et al., 2010). In task-switching, participants are typically asked to perform one of two tasks cued on a trial-by-trial basis. Trials where participants need to switch task typically suffer from a *switch cost* [i.e., lower accuracy and longer response time (RT)] when compared to consecutive trials using the same task. System-switching would be a special case of task-switching in which each task relies on a different categorization system. To explore this possibility, Crossley et al. (2017) also included a condition in which participants needed to switch between two different declarative strategies. The results showed that the switch cost was smaller when switching within the declarative system compared to switching between a declarative and a procedural system.

### 1.2. Practice and preparation in system-switching

One of the important findings in the task-switching literature is that the switch cost can be reduced by introducing a delay between the presentation of an external cue indicating the task to be performed in the upcoming trial and stimulus presentation (for reviews, see Kiesel et al., 2010; Vandierendonck et al., 2010). This delay allows for *preparation*, and preparation has been shown to reduce, but not eliminate, RT switch costs and sometime also reduce the accuracy switch cost (Vandierendonck et al., 2010). A number of hypotheses have been proposed to account for the reduction in switch cost. For example, Koch (2003) suggested that preparation time is used to facilitate preparatory retrieval of task-specific stimulus-response rules (see also Altmann, 2004). Alternatively, preparation time can be used to reduce the carry-over activity from the task performed in the previous trial (Vandierendonck et al., 2010). While neither one of these explanations can fully account for all the observed task-switching results, especially the residual switch cost (Kiesel et al., 2010), both would suggest that preparation time should facilitate categorization system-switching as measured by a higher proportion of participants identified as switchers and a reduced switch cost. In the former case, preparation time could be used to select the new categorization system needed for the upcoming trial. In the latter case, preparation time could be used to disengage the categorization system used in the previous trial.

In Crossley et al. (2017), a larger proportion of switchers was identified by increasing the number of training trials in the experimental session. The task-switching literature has also found some positive effects of training on task-switching, although the switch cost was not significantly reduced. For example, Minear and Shah (2008) found reduced RT on trials following switches for both switch and non-switch trials, suggesting faster recuperation from switches after practice. In addition, Logan and Schneider (2006) showed that the congruency of the cue and target had a smaller effect with transparent cues after extensive practice. The cue is nontransparent in categorization system-switching experiments (i.e., the background color is arbitrarily associated with the categorization tasks), but it is worth noting that participants need to learn the tasks using trial-and-error learning. This differs from most task-switching experiments, in which participants are asked to make judgments such as odd/even or large/small, i.e., tasks that they already know how to do before the beginning of the experiment. In contrast, participants are not familiar with the categories used in perceptual categorization experiments and they need to learn how to group abstract stimuli. As a result, the individual tasks are more difficult and might benefit from the additional training. Intuitively, it may be easier to switch between two well-known tasks than between two unfamiliar tasks. Given this evidence and reasoning, we hypothesized that adding a second training session should facilitate system-switching by increasing the proportion of participants identified as switchers. The task-switching literature suggests, however, that switch costs should not be much affected by the additional practice.

## 2. Experiment

The main goal of this experiment was to identify factors influencing the proportion of participants who successfully learn to switch between a procedural and a declarative categorization system on a trial-by-trial basis. Toward this goal, we trained participants in one of four conditions: (1) 1 session without preparation time (1S/NOPREP), (2) 1 session with preparation time (1S/PREP), (3) 2 sessions without preparation time (2S/NOPREP), and (4) 2 sessions with preparation time (2S/PREP). Note that 1S/NOPREP is similar to previous work on system-switching (e.g., Erickson, 2008; Ashby and Crossley, 2010; Crossley et al., 2017) and served as a control condition.

### 2.1. Methods

#### 2.1.1. Participants

One hundred thirty-three participants were recruited from the Purdue University undergraduate population to participate in this experiment. Participants were randomly assigned to one of four conditions: 1S/NOPREP (*n* = 34), 1S/PREP (*n* = 27), 2S/NOPREP (*n* = 37), and 2S/PREP (*n* = 35). Each participant was given credit for participation as partial fulfillment of a course requirement. Participants gave written informed consent and all procedures were approved by the Purdue University Human Research Protection Program Institutional Review Board, protocol #1209012631.

#### 2.1.2. Material

**Figure 1:**
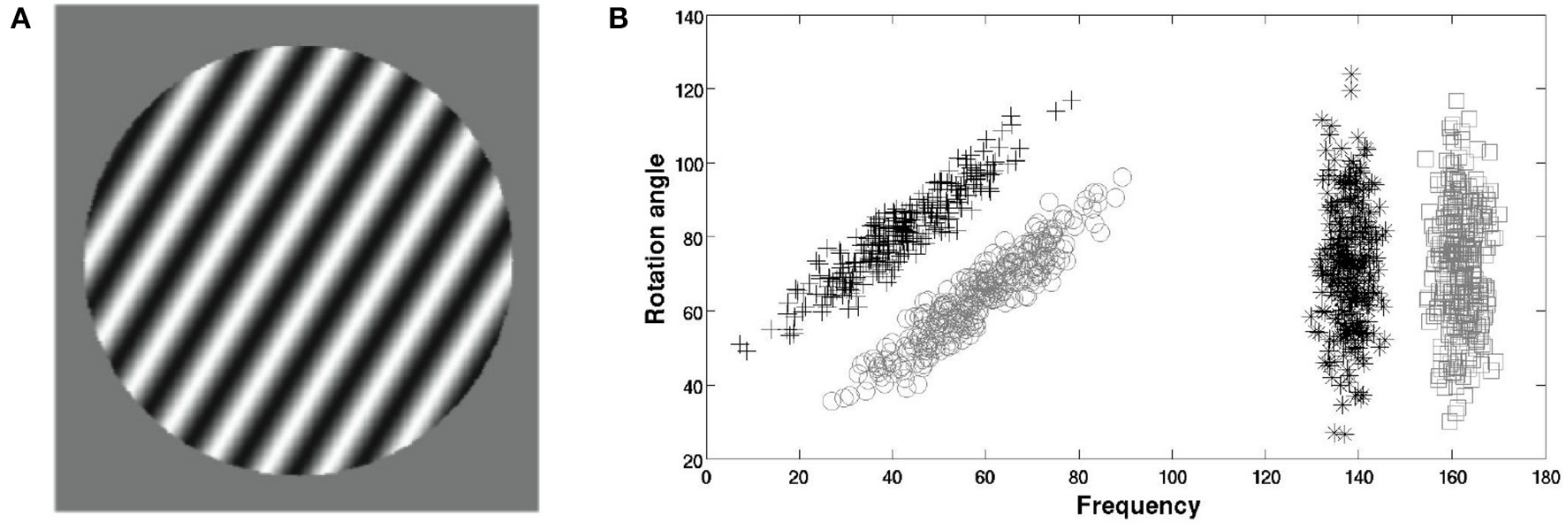
Stimuli used in the experiment. **(A)** An example stimulus. **(B)** Category structures. “+” denote members of category “A,” “◦” denote members of category “B,” “^*^” denote members of category “C,” and “□” denote members of category “D.”

The stimuli were circular sine-wave gratings of constant contrast and size presented on a 21-inch monitor (1,920 × 1,080 resolution). Each stimulus was defined in a 2D space by a set of points (*frequency, orientation*) where *frequency* (bar width) was calculated in cycles per degree (cpd), and *orientation* (counterclockwise rotation from horizontal) was calculated in radian. The stimuli were generated with Matlab using Brainard's (1997) Psychophysics toolbox and occupied an approximate visual angle of 5°. In each trial, a single stimulus was presented in the center of the screen. Figure 1A shows an example stimulus.

The stimuli were separated into four categories generated into an arbitrary 200 × 100 coordinate system using the randomization technique of Ashby and Gott (1988). Figure 1B shows the categories which were arbitrarily labeled with letters A–D from left to right. The category A and B structures were II and were generated using bivariate normal distributions: μ_*A*_ = (42, 80), $\Sigma_{A}=\left(\begin{array}{cc} 145 & 135\\ 135 & 145 \end{array}\right)$, μ_*B*_ = (58, 64), Σ_*B*_ = Σ_*A*_. The category C and D structures were RB and were generated using bivariate normal distributions: μ_*C*_ = (138, 72), $\Sigma_{C}=\left(\begin{array}{cc} 10 & 0\\ 0 & 280 \end{array}\right)$, μ_*D*_ = (162, 72), and Σ_*D*_ = Σ_*C*_. The stimulus arbitrary coordinate system was then re-scaled into a *frequency* × *orientation* space using a nonlinear transformation (Crossley et al., 2017; see **Appendix**). This yielded stimuli ranging in frequency from 0.29 to 8.6 cpd and from 34 to 95° in orientation (counterclockwise from horizontal). Perfect accuracy was possible and optimal performance required responding to the A–B stimuli using a procedural system and responding to the C–D stimuli with a declarative system.

Stimulus presentation, feedback, and response recording were controlled and acquired using Matlab. The screen background color was used as a cue to inform participants about the possible responses. RB stimuli (C–D) were presented with a blue background while II stimuli (A–B) were presented with a green background. Responses were given on a standard keyboard: the “s” key was used for category A, the “d” key was used for category B, the “k” key was used for category C, and the “l” key was used for category D. The response keys were covered with blank stickers, and the category labels (A–D) were displayed at the bottom of the screen in an order mapping the response buttons on the keyboard. After each response was made, auditory feedback was presented: a high pitch tone for a correct response, a buzzsaw sound for an incorrect response, and a distinctive two note sound for an incorrect response key.

#### 2.1.3. Procedure

For the 1S/NOPREP and 1S/PREP conditions, the experiment lasted 1 session. The experimental session was divided into 7 blocks of 100 trials (for a total of 700 trials). In Block 1, participants were trained only in RB categorization (C–D). In Blocks 2–5, participants were trained only in II categorization (A–B). II categorization training was made longer based on prior results showing that II categorization is typically more difficult to learn than RB categorization (e.g., Hélie et al., 2010; Hélie and Ashby, 2012). In Block 6, RB and II trials were randomly interleaved, so optimal performance required participants to switch categorization system on a trial-by-trial basis. Finally, Block 7 was similar to Block 6, except that the response buttons were switched, i.e., the “s” key was now used for category B, the “d” key was now used for category A, the “k” key was now used for category D, and the “l” key was now used for category C. This procedure has been shown to affect the procedural system (used for II categorization) more than the declarative system (used for RB categorization) (Ashby et al., 2003). During Block 7, the response labels at the bottom of the screen were changed appropriately (so they now read B, A, D, C).

For the 2S/NOPREP and 2S/PREP conditions, the experiment lasted 2 sessions scheduled during the same week. Each session was divided into 7 blocks of 100 trials (700 trials per session, 1,400 trials total). Session 1 was identical to the session described above for the 1S/NOPREP and 1S/PREP conditions, except that Block 7 was not a button-switch. Instead, Block 7 used randomly interleaved RB and II trials (same as Block 6). Session 2 was identical to the session described above for the 1S/NOPREP and 1S/PREP conditions, including a button-switch in Block 7.

In all condition, a trial went as follows: a fixation point (crosshair) appeared on the screen for 1,500 ms. In the 1S/NOPREP and 2S/NOPREP conditions, the crosshair background was gray (neutral). In the 1S/PREP and 2S/PREP conditions, the crosshair background was either blue or green, cuing whether the following stimulus was going to be RB (C–D) or II (A–B). This 1,500 ms cue was the preparation time. Next, the crosshair disappeared and was immediately replaced by a stimulus. In all conditions, the stimulus background was indicative of the possible responses (blue for C–D; green for A–B). When the participants made a response or after 5 s had elapsed, the stimulus disappeared and auditory feedback was presented. The participants were allowed to take a break between blocks if they wished.

The participants were told that they were taking part in a categorization experiment and that they needed to learn the categories using trial-and-error. The participants were also informed before the experiment that stimuli presented on a blue background were either from the category C or D, and that stimuli presented on a green background were either from the A or B category. Prior to beginning Block 1 (in both sessions), participants were told that the relevant stimulus feature to determine category membership in the following block was bar width. After completing Block 1, participants were told that bar width and orientation were both relevant features for categorization in the following blocks (also in both sessions). Finally, participants were notified about the button switch before beginning a button switch block (Block 7 in Sessions 1 or 2 depending on the condition).

### 2.2. Results

**Figure 2:**
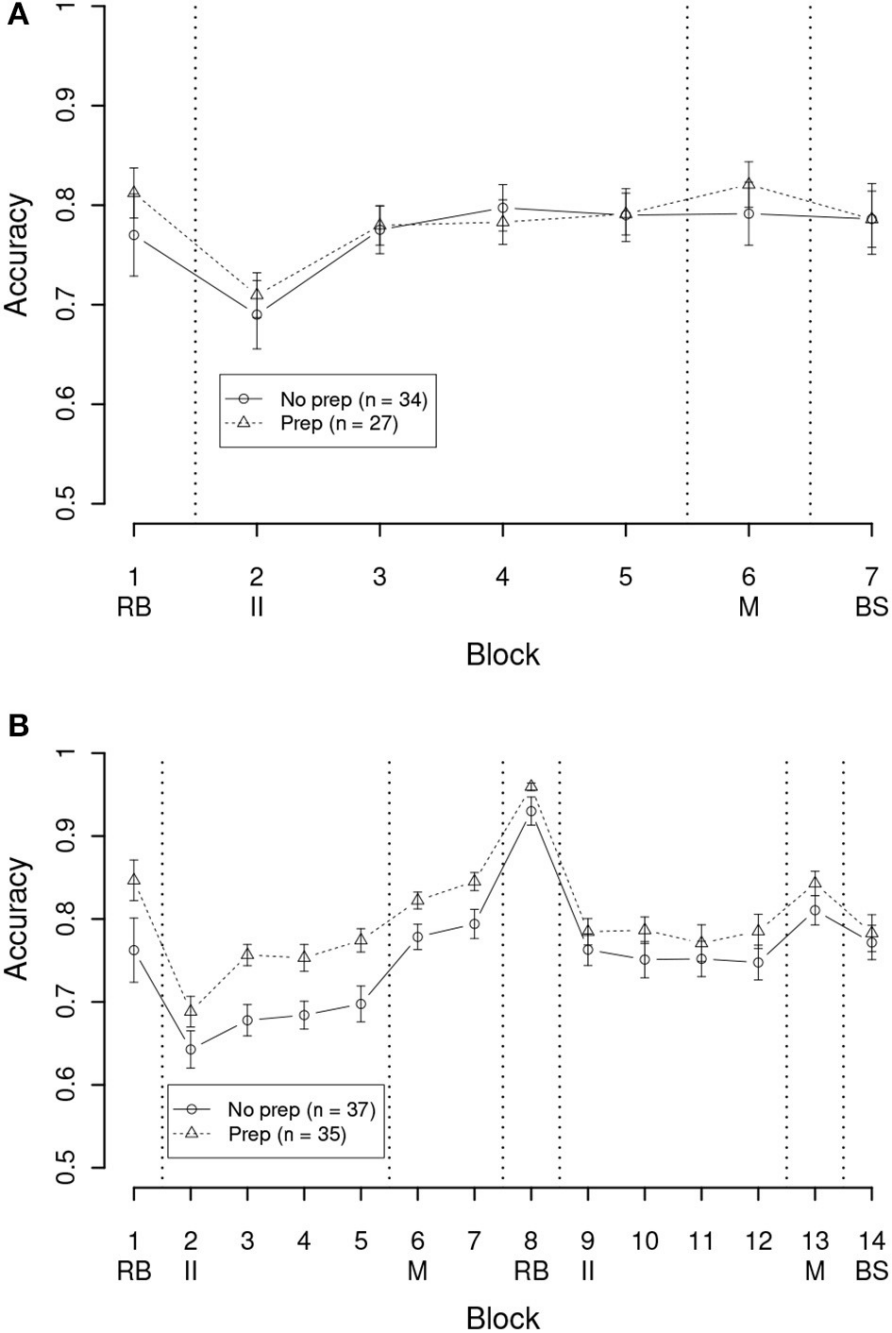
Mean accuracy per block in the experiment. Vertical dashed lines indicate a change of trial type, and the letters under the block numbers indicate the beginning block of a trial type: RB, Rule-based; II, Information-integration; M, Mixed trials (interleaved); BS, Button-switch trials. **(A)** Conditions with 1 training session. **(B)** Conditions with 2 training sessions. Error bars are between-subject standard error of the mean.

The mean accuracy in each block for each condition is shown in Figure 2. First let us consider the 1-session conditions (Figure 2A). As can be seen, participants performed well in the initial RB training block, and were also able to learn the II stimuli. Further, intermixing the trials and the button-switch manipulation had no effect on overall accuracy. Lastly, there was no apparent difference between the 1S/PREP and 1S/NOPREP conditions, suggesting that preparation time did not allow for more accurate categorization. These observations were supported by a Preparation time (between, PREP vs. NOPREP) × Block (within, 1–7) mixed ANOVA. The effect of Block reached statistical significance [*F*_(6, 354)_ = 7.73, *p* < 0.001, η^2^ = 0.51], showing that the maximum mean accuracy (Block 6, 80.4%) was higher than the minimum mean accuracy (Block 2, 70.0%). Both the effects of Preparation time [*F*_(1, 59)_ = 0.12, *n.s.*, η^2^ = 0.00] and the Block × Preparation time interaction [*F*_(6, 354)_ = 0.60, *n.s.*, η^2^ = 0.09] failed to reach statistical significance.

Let us now turn to the 2-sessions conditions (Figure 2B). As can be seen, accuracy in the first session (Blocks 1–7) was similar to that in the 1-session conditions (Figure 2A). In the second session (Blocks 8–14), the participants did better in the RB training block, but performed similarly in the II training blocks and the intermixed block. However, unlike in the 1-session conditions, there was a small reduction in accuracy in the button-switch block (Block 14). Another difference between the 1- and 2-sessions conditions is that participants who had preparation time (2S/PREP) were more accurate than participants without preparation (2S/NOPREP). These observations were confirmed by a Preparation time (between, PREP vs. NOPREP) × Block (within, 1–14) mixed ANOVA. First, as in the 1-session conditions, the effect of Block reached statistical significance $\left[F_{\left(13,\;910\right)}=41.25,p<0.001,\eta^{2}=0.90\right]$, showing that the maximum mean accuracy (Block 8, 94.4%) was higher than the minimum mean accuracy (Block 2, 66.5%). However, unlike in the 1-session conditions, the effect of Preparation time also reached statistical significance $\left[F_{\left(1,\;70\right)}=5.59,p<0.05,\eta^{2}=0.07\right]$. Mean accuracy for the 2S/PREP condition was 80.0% while mean accuracy for the 2S/NOPREP condition was 75.5%. Finally, the Preparation time × Block interaction failed to reach statistical significance $\left[F_{\left(13,\;910\right)}=1.35,n.s.,\eta^{2}=0.31\right]$.

#### 2.2.1. Model-based analyses

Participants who can or cannot switch system on a trial-by-trial basis are typically identified using model-based analysis (Erickson, 2008; Ashby and Crossley, 2010; Crossley et al., 2017). Specifically, one selects the data from the last intermixed trials block (identified with a “M” in Figure 2) and separates RB from II trials. Decision bound models (Ashby, 1992; Maddox and Ashby, 1993) are then fit separately to the RB and II data. There are three general classes of decision bound models, namely guessing models, explicit-reasoning models, and procedural-learning models (Hélie et al., 2017). For each data set, the best model is selected using the Bayes information criterion (Hélie, 2006). Participants whose data are best-fit by the optimal models are labeled as “switchers.” All other participants are labeled as “non-switchers.” In the current experiment, the optimal decision bound model for the RB data was an explicit-reasoning model (i.e., a unidimensional rule on the *x*-axis of Figure 1B) and the optimal decision bound model for the II data was a procedural-learning model (i.e., the general linear classifier). More details on decision bound models and fitting procedures can be found in Maddox and Ashby (1993) or Hélie et al. (2017).

**Figure 3:**
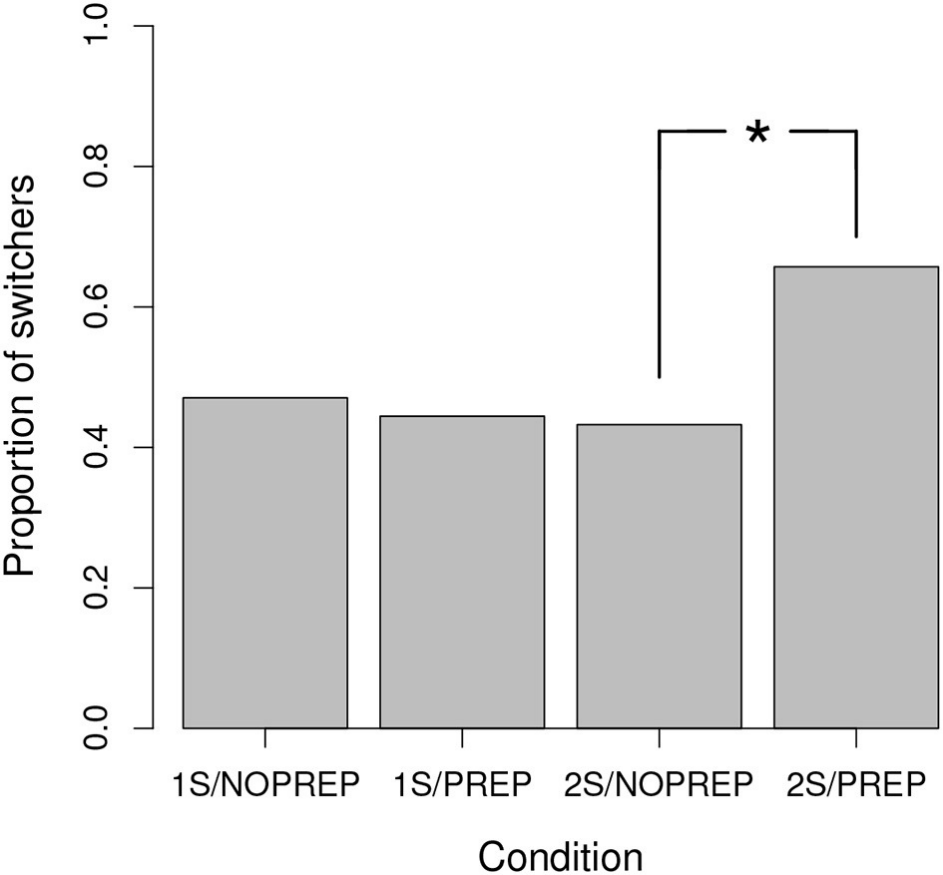
Proportion of switchers in each condition. ^*^Denotes a proportion statistically significant (*p* < 0.05).

The proportion of participants identified as switchers in each condition is shown in Figure 3. As can be seen, 47.1% of the participants were identified as switchers in the 1S/NOPREP condition, which is in line with the proportion of switchers identified in previous work (Crossley et al., 2017). Figure 3 also shows that adding preparation time (1S/PREP) or adding a second training session (2S/NOPREP) did not increase the proportion of switchers (44.4% and 43.2%, respectively). However, adding preparation time in the 2-session condition (2S/PREP) increased the proportion of participants who could switch from trial-to-trial to 65.7%. This proportion is substantially higher than the proportion of switchers reported in previous work (Erickson, 2008; Ashby and Crossley, 2010; Crossley et al., 2017). A one-tailed test of two independent proportions for binomial data derived from the generalized likelihood criterion (Larsen and Marx, 2006, p. 578) showed that the proportion of switchers was higher in the 2S/PREP than in the 2S/NOPREP condition (*Z* = 1.91, *p* < 0.05). In contrast, the proportion of switchers did not differ between the 1S/PREP and 1S/NOPREP conditions (*Z* = 0.20, *n*.*s*.).

#### 2.2.2. Trial-by-trial switch cost (accuracy)

One well-known effect in the task switching literature is switch cost (Kiesel et al., 2010). Essentially, when tasks are alternating, there is a cost associated with the switch that is visible in accuracy and RT. Crossley et al. (2017) argued that system-switching could be interpreted as a form of task-switching. To explore this possibility, the following analyzes focused on the last intermixed trial block in each condition. For each participant, the first trial of the block was dropped, and each subsequent trial was labeled as “switch” or “stay” depending whether the previous trial required using the same system (or not) to perform optimally.

**Figure 4:**
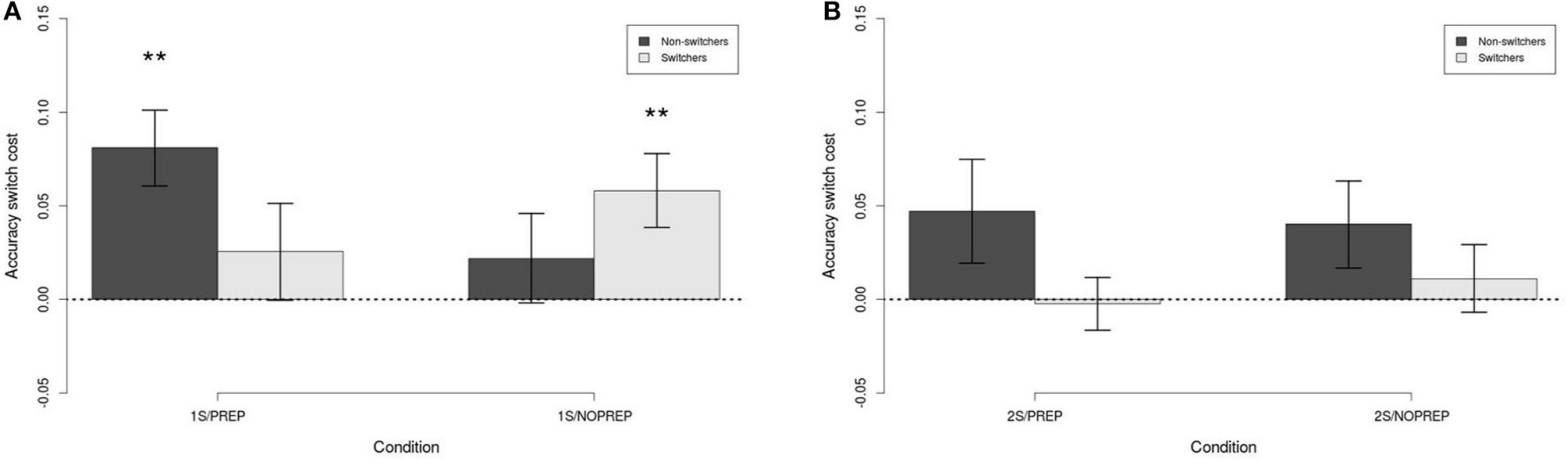
Accuracy switch cost in each condition. **(A)** Block 6 in 1-session conditions. **(B)** Block 13 in 2-sessions conditions. Error bars are between-subject standard error of the mean. ^**^p < 0.01.

Figure 4 shows the accuracy switch cost for switchers and non-switchers in each condition (i.e., *stay–switch*). A separate Switch (between, Switchers vs. Non-switchers) × Preparation time (between, PREP vs. NOPREP) ANOVA was performed for 1- and 2-sessions conditions. First, Figure 4A shows the switch cost in Block 6 for the 1-session conditions. The interaction between the factors was statistically significant $\left[F_{\left(1,\;57\right)}=4.03,p<0.05,\eta^{2}=0.07\right]$, but both main effects failed to reach statistical significance [both $F_{\left(1,\;57\right)}<1,n.s.,\eta^{2}<0.01]$. Decomposing the effect of Preparation time within each level of Switch showed that the effect of Preparation time was trending towards statistical significance for non-switchers $\left[t_{\left(31\right)}=1.88,p<0.10,\eta^{2}=0.10\right]$ but not for switchers $\left[t_{\left(22\right)}=-1.00,n.s.,\eta^{2}=0.04\right]$. Hence, the interaction was likely caused by the crossover in switch cost, but the individual conditions did not statistically differ from each other.

Another informative way to analyze the data was to compute whether the switch cost is statistically different from 0. One-sample *t*-tests were performed for each condition, and the results showed that the switch cost was statistically significant for non-switchers with preparation time $\left[t_{\left(14\right)}=4.00,p<0.01,\eta^{2}=0.53\right]$ and for switchers with no preparation time $\left[t_{\left(15\right)}=2.95,p<0.01,\eta^{2}=0.37\right]$. The other two conditions did not have a statistically significant switch cost (both *t* < 1, *n*.*s*., η^2^ < 0.09). Hence, for the 1-session conditions, the absence of preparation time resulted in a switch cost for switchers (as expected), while the presence of preparation time produced a switch cost for non-switchers (more later).

Next, Figure 4B shows the accuracy switch cost for the 2-sessions conditions in Block 13. Unlike for the 1-session conditions, the ANOVA showed no effect of Preparation time $\left[F_{\left(1,\;68\right)}=0.03,n.s.,\eta^{2}=0.00\right]$, Switch $\left[F_{\left(1,\;68\right)}=3.49,n.s.,\eta^{2}=0.05\right]$, or Preparation time × Switch interaction $\left[F_{\left(1,\;68\right)}=0.24,n.s.,\eta^{2}=0.00\right]$. In addition, none of the conditions showed a switch cost on accuracy (all *t* < 1.73, *n*.*s*., η^2^ < 0.21). Hence, these results showed no evidence of an accuracy switch cost in the 2-session conditions.

#### 2.2.3. Trial-by-trial switch cost (response time)

**Figure 5:**
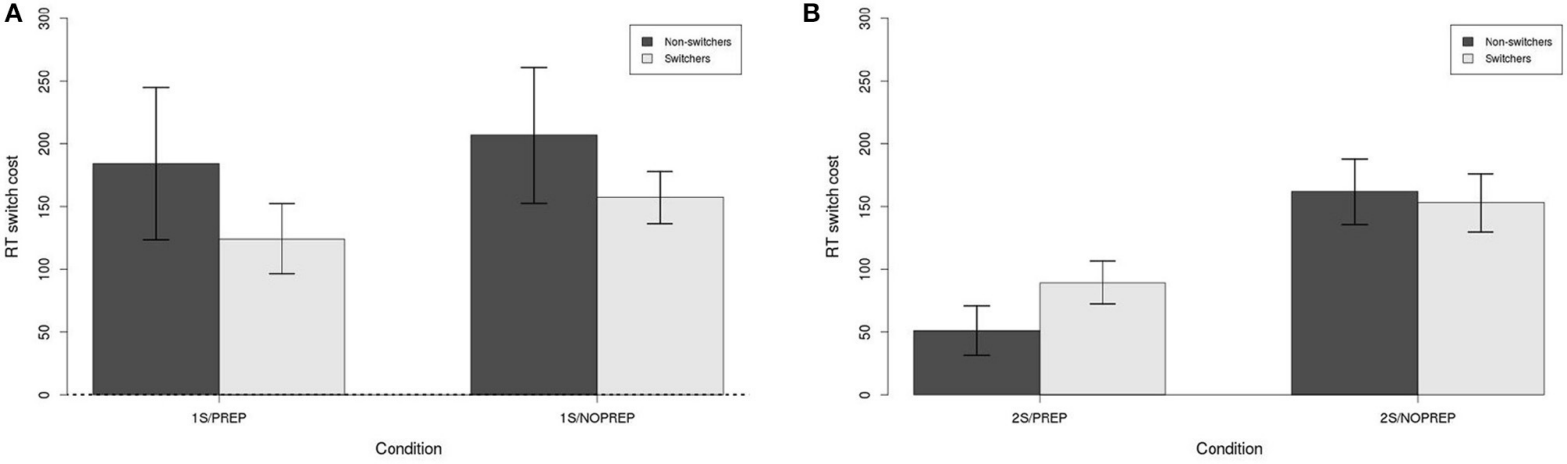
Switch cost (RT) in each condition. **(A)** Block 6 in 1-session conditions. **(B)** Block 13 in 2-sessions conditions. All switch costs in all panels are statistically significant (*p* < 0.05). Error bars are between-subject standard error of the mean.

Figure 5 shows the RT switch cost for switchers and non-switchers in each condition (i.e., *switch–stay*). Similar to accuracy, a separate Switch (between, Switchers vs. Non-switchers) × Preparation time (between, PREP vs. NOPREP) ANOVA was performed for 1- and 2-sessions conditions. First, Figure 5A shows the switch cost in Block 6 for the 1-session conditions. None of the effects were statistically significant, with no evidence of manipulation effect [all $F_{\left(1,\;57\right)}<1.35,n.s.,\eta^{2}<0.03]$. However, all the switch costs were statistically greater than zero (all *t* > 3.05, *p* < 0.01, η^2^ > 0.39). The mean switch cost was 168 ms. Hence, for the 1-session conditions, a RT switch cost was present but no difference was found between switchers and non-switchers, or with or without preparation time.

Next, Figure 5B shows the RT switch cost for the 2-sessions conditions in Block 13. The ANOVA showed a statistically significant effect of Preparation time $\left[F_{\left(1,\;68\right)}=14.23,p<0.001,\eta^{2}=0.17\right]$, but no effect of Switch $\left[F_{\left(1,\;68\right)}=0.41,n.s,\eta^{2}=0.01\right]$ and no Preparation time × Switch interaction $\left[F_{\left(1,\;68\right)}=1.03,n.s.,\eta^{2}=0.02\right]$. The mean switch cost without preparation time was 158 ms, but reduced to 70 ms with preparation time. Still, even with the reduction of switch cost caused by preparation time, residual switch costs in all conditions were statistically greater than 0 (all *t* > 2.63, *p* < 0.05, η^2^ > 0.38). Hence, preparation time helped with reducing RT switch costs, but only after sufficient practice.

#### 2.2.4. Button-switch interference

Previous research has shown interference (i.e., lower accuracy) when changing the location of response buttons after learning II but not RB categories (Ashby et al., 2003), so it is reasonable to expect a similar pattern for participants identified as switchers (Crossley et al., 2017). Button-switch interference is calculated by separately subtracting the accuracy in RB and II trials in the button-switch block from the corresponding trials in the last intermixed block (Block 6 and 13 for the 1- and 2-sessions conditions, respectively).

**Figure 6:**
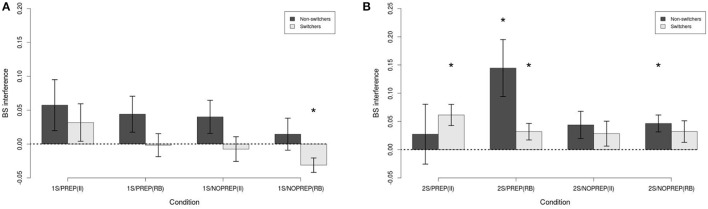
Button-switch interference. **(A)** 1-session conditions. **(B)** 2-sessions conditions. ^*^p < 0.05.

The button-switch interference for the 1-session conditions is shown in Figure 6A. As can be seen, the interference was fairly small in all conditions. A Switch (between, Switchers vs. Non-switchers) × Preparation time (between, PREP vs. NOPREP) × Categories (within, RB vs. II) mixed ANOVA showed a statistically significant effect of Switch $\left[F_{\left(1,\;57\right)}=4.29,p<0.05,\eta^{2}=0.07\right]$. The mean button-switch interference for non-switchers was 0.04. In contrast, the mean interference for switchers was −0.01. All other effects and interactions were non-significant [all $F_{\left(1,\;57\right)}<2.62,n.s.,\eta^{2}<0.05]$. The only condition that had interference statistically different from 0 was in the 1S/NOPREP(RB)/Switchers condition $\left[t_{\left(15\right)}=-2.94,p<0.05,\eta^{2}=0.37\right]$. Note that this interference was negative, indicating a facilitation effect. All other interference did not statistically differ from 0 (all |*t*| < 1.67, *n*.*s*., η^2^ < 0.17).

The button-switch interference for the 2-session conditions is shown in Figure 6B. As can be seen, the interference was again fairly small in all conditions except for the 2S/PREP(RB)/Non-switchers condition. A Switch (between, Switchers vs. Non-switchers) × Preparation time (between, PREP vs. NOPREP) × Categories (within, RB vs. II) mixed ANOVA showed a statistically significant three-way interaction $\left[F_{\left(1,\;68\right)}=4.31,p<0.05,\eta^{2}=0.06\right]$ as well as a significant two-way Switch × Categories interaction $\left[F_{\left(1,\;68\right)}=4.18,p<0.05,\eta^{2}=0.06\right]$. All other effects were not statistically significant [all $Fs_{\left(1,\;136\right)}<2.45,n.s.,\eta^{2}<0.04]$. Based on the hypothesis that switchers and non-switchers should have a different pattern of button-switch interference, we decomposed the three-way interaction to explore the effects of Preparation time × Categories within each level of Switch. The decomposition showed no statistically significant effect for switchers [all $F_{\left(1,\;37\right)}<1,n.s.,\eta^{2}<0.03]$ and non-switchers [all $F_{\left(1,\;31\right)}<3.79,p<0.10,\eta^{2}<0.11]$. As a result, we turned to decomposing the two-way interaction. Because we expected button-switch interference to affect RB and II categories differently, we investigated the effect of Categories within each level of Switch. Again, the effect of Categories was not statistically significant for switchers $\left[F_{\left(1,\;38\right)}<1,n.s.,\eta^{2}=0.02\right]$ and non-switchers $\left[F_{\left(1,\;32\right)}=2.07,n.s.,\eta^{2}=0.06\right]$. Hence, the ANOVA showed that the factors interacted in affecting button-switch interference, but there was no clear effect of individual factors for switchers and non-switchers.

Comparing the interference with 0 may facilitate understanding the ANOVA. Four conditions showed statistically significant button-switch interference: (1) 2S/PREP(RB)/Non-switchers $\left[t_{\left(11\right)}=2.86,p<0.05,\eta^{2}=0.43\right]$, (2) 2S/PREP(RB)/Switchers [*t*_(22)_ = 2.18, *p* < 0.05, η^2^ = 0.18], (3) 2S/NOPREP(RB)/Non-switchers [*t*_(20)_ = 3.09, *p* < 0.01, η^2^ = 0.32], and (4) 2S/PREP(II)/Switchers [*t*_(22)_ = 3.27, *p* < 0.01, η^2^ = 0.33]. All other interference did not statistically differ from 0 (all t < 1.82, n.s., η^2^ < 0.15). Together, these results suggest that while practice and preparation time jointly increased the proportion of switchers and reduced the trial-by-trial RT switch cost, this came at the cost of an increased button-switch interference: (1) Without extended practice, none of the conditions showed significant button-switch interference. (2) With practice, preparation time produced button-switch interference in all conditions except for non-switchers categorizing II stimuli. (3) With practice but without preparation time, no button-switch interference was observed, except for non-switchers categorizing RB stimuli.

## 3. General discussion

This article explored the effects of preparation and practice on system-switching in categorization. The experiment trained participants on RB and II categories first separately (using a blocked procedure) and then using intermixed blocks. In both the blocked and intermixed trials, the type of categories (RB or II) was cued using a different background color. The experiment ended with a block of intermixed trials where the locations of the response buttons were switched.

Four different conditions were run: (1) 1 training session with no preparation time (1S/NOPREP), (2) 1 training session with preparation time (1S/PREP), (3) 2 training sessions with no preparation time (2S/NOPREP), and (4) 2 training sessions with preparation time (2S/PREP). Model-based analyses were run to identify each participant's strategy (Hélie et al., 2017), and trial-by-trial switch costs (both on accuracy and RT) and button-switch interference were calculated. Overall, the results suggest that practice and preparation time can be beneficial to system-switching, but only when they are jointly present. Specifically, the 2S/PREP condition had a larger proportion of switchers, no evidence of trial-by-trial accuracy switch cost, and a smaller trial-by-trial RT switch cost (when compared with 2S/NOPREP). However, these benefits came with button-switch interference for all but the non-switchers categorizing II stimuli. A summary of the obtained results is shown in Table 1. We now discuss the individual effects of preparation time and practice as well as other relevant findings from the experiment.

**Table 1:** Summary results of the experiment.

**Condition**	**Switch cost (acc.)**		**Switch cost (rt)**		**BS interference**	
	**S**	**NS**	**S**	**NS**	**S**	**NS**
1S/NOPREP	Y	N	Y	Y	Y/N	N/N
1S/PREP	N	Y	Y	Y	N/N	N/N
2S/NOPREP	N	N	Y	Y	N/N	Y^†^/N
2S/PREP	N	N	Y	Y	Y/Y	Y/N

*Y, Yes; N, No; S, Switchers; NS, Non-switchers. For BS interference, RB/II. †Negative interference, i.e., facilitation*.

### 3.1. The effect of preparation time on system-switching

There is an extensive literature on the effect of preparation time in task-switching (for reviews, see, e.g., Kiesel et al., 2010; Vandierendonck et al., 2010). Generally, preparation time reduces RT and accuracy trial-by-trial switch costs. It is thus reasonable to expect a similar effect on system-switching. However, when not paired with extended training, preparation time only had a small effect on system-switching. First, there was no evidence that preparation time alone could increase the proportion of switchers (1S/NOPREP vs. 1S/PREP). Second, there was no evidence that preparation time alone could reduce the RT trial-by-trial switch cost. However, preparation time did eliminate the accuracy trial-by-trial switching cost. This gain from preparation time was however restricted to switchers. For non-switchers, the accuracy trial-by-trial switch cost was only present for participants with preparation time. Finally, preparation time did eliminate the button-switch interference of switchers categorizing RB stimuli (which was present in the 1S/NOPREP condition). Hence, preparation time seems to have a positive effect on accuracy switch cost, but this effect is only present for switchers. For non-switchers, preparation time has either no effect or produced an accuracy switch cost. Surprisingly, preparation time alone did not affect RT switch costs.

In perceptual categorization, the preparation time could have been used either to select the appropriate categorization system for the upcoming trial or to disengage from the categorization system used in the previous trial (Kiesel et al., 2010; Vandierendonck et al., 2010). In line with task-switching research, system-switching could be interpreted in terms of an abstract rule (or rule set) that associates the categorization systems with the task cues. Within this framework, the former would correspond to rule selection while the later would correspond to rule switching (Ashby et al., 2005). In COVIS (Ashby et al., 1998), rule selection and rule switching are different cognitive operations relying on different brain mechanisms. These brain mechanisms, form a circuit and work collaboratively so they are difficult to disentangle in neurologically-intact participants (Hélie et al., 2012a,b). It is possible, however, that the circuit needs training, which would explain why preparation time required additional practice in order to be beneficial. Disentangling whether the benefits of preparation time depend on switching or selection is outside the scope of the present article as it would require either studying specific patient populations with selective deficits or disrupting neural signaling in neurologically-intact participants (e.g., with transcranial magnetic stimulation). One promising candidate approach would be to use more than two tasks (Kleinsorge and Scheil, 2015) and to calculate the number of task confusion errors (Steinhauser and Gade, 2015).

### 3.2. The effect of practice on system-switching

Unlike preparation time, the effect of practice on task-switching has only been studied sporadically (Logan and Schneider, 2006; Minear and Shah, 2008). While both studies have found some positive effects of practice, neither one of them has found a significant reduction in switch cost. The results in our experiment are partially consistent with those earlier studies. First, practice alone (without added preparation time) did not numerically increase the proportion of switchers. Second, participant accuracy improved in the second session for RB stimuli but not for II stimuli during the blocked trials. This suggests that participants may have reached asymptotic accuracy with the II categories used in the present experiment, and that additional training did not improve performance in the individual categorization tasks (as hypothesized). Third, similar to Logan and Schneider (2006) and Minear and Shah (2008), practice did not affect the RT trial-by-trial switch cost. However, practice did reduce the accuracy trial-by-trial switch cost, at least for participants identified as switchers. Similar to preparation time, practice removed the button-switch interference present for switchers categorizing RB stimuli, but in addition produced facilitation for non-switchers categorizing RB stimuli. Again, practice alone had no effect on trial-by-trial RT switch costs, but it helped reduce switchers' trial-by-trial accuracy switch cost and both switchers' and non-switchers' button-switch interference.

### 3.3. Implications for multiple-systems theories of category learning

This research further adds to previous evidence that system-switching on a trial-by-trial basis is extremely difficult (Erickson, 2008; Ashby and Crossley, 2010; Crossley et al., 2017). However, a number of factors borrowed from task-switching could make system-switching more likely. While preparation time and practice separately only had small effects on system-switching, combining them significantly increased the proportion of switchers, eliminated the accuracy switch cost, and reduced by half the RT switch cost. It is possible that switching between the categorization systems is a separate task that takes time (hence the effect of preparation time) and can be trained (hence the effect of practice). The preparation time included in the experiment may have been initially too short, but as participants became more proficient at system-switching it may have become sufficient, which would explain the combined effects of preparation time and practice. If this is the case, then models including multiple categorization systems would need an additional switching system that is trainable, and this system could take the form of a more abstract set of rules. The current experiment was not designed specifically to test this hypothesis, but future work should be devoted to dissociating training on the categories themselves from training at system-switching.

### 3.4. Future work and limitations

This manuscript described the effects of two factors known to facilitate task-switching on system-switching. The facilitating effect found in task-switching seemed to be also present in the system-switching experiment. This suggests that the literature on system-switching may benefit from testing other factors that facilitate task-switching (e.g., the effects of cue encoding and verbal mediation). Another limitation of the present experiment is linked to the labeling of switchers and non-switchers. Here we used the same terminology as previous work (Erickson, 2008; Ashby and Crossley, 2010; Crossley et al., 2017), and labeled all participants who were not best fit by optimal models as non-switchers. However, one implication is that non-switchers are a mixed bag of participants who truly did not switch, but also participants who switched between two non-optimal strategies (e.g., switching between two declarative strategies). In the later case, non-switchers is a misnomer: *suboptimal switchers* might be a more appropriate label. Future research should try to distinguish between non-switchers and suboptimal switchers, which may help explain some counter-intuitive results observed for non-switchers. For example, the presence of interference for non-switchers in the 1S/PREP condition is puzzling and may have been caused by a number of potentially different mechanisms. One possibility is that non-switchers tried to switch during the preparation time, but failed. Some participants may also have switched between two suboptimal strategies, which would have made them suboptimal switchers. In contrast, non-switchers could have not even tried to switch when there was no preparation time, which would have made them *true* non-switchers. As a result, the label *non-switchers* may refer to different types of participants with and without preparation time. Unfortunately, the present experiment was not designed to directly test this possibility, so it is difficult at this point to confirm or eliminate this interpretation. It is encouraging that work is currently being done to begin to understand the interaction between the different categorization systems, and much more data is needed so that a solid understanding of system-switching can emerge.

## Ethics statement

This study was carried out in accordance with the recommendations of the Purdue University Social Science Institutional Review Board with written informed consent from all subjects. All subjects gave written informed consent in accordance with the Declaration of Helsinki. The protocol was approved by the Purdue University Social Science Institutional Review Board.

## Author contributions

The author confirms being the sole contributor of this work and approved it for publication.

### Conflict of interest statement

The author declares that the research was conducted in the absence of any commercial or financial relationships that could be construed as a potential conflict of interest.

## Appendix

This Appendix describes the transformation used to generate the spatial frequency and orientation defining the stimuli used in the experiment. The transformation is based on results presented in Treutwein et al. (1989) and was first presented in Crossley et al. (2017).

First, the arbitrary coordinates (*x, y*) described in section 2.1.2 were linearly transformed into (*x*_*T*_, *y*_*T*_) pairs to span the interval (−0.8, 4.2) on dimension *x*_*T*_ and (0.6, 1.8) on dimension *y*_*T*_:

(A.1) $$\begin{array}{l} x_{T}=\frac{3x}{100}-1 \end{array}$$

(A.2) $$\begin{array}{l} y_{T}=\frac{3\pi }{8}\frac{y}{100}+\frac{\pi }{11} \end{array}$$

Second, the (*x*_*T*_, *y*_*T*_) values were transformed into their respective measurement space. For *x*_*T*_, the values were mapped to spatial frequency *f* via:

(A.3) $$\begin{array}{l} f=2^{x_{T}} \end{array}$$

This completes the transformation of the *x* dimension into a frequency expressed in cpd.

To convert *y*_*T*_ into orientation, all *y*_*T*_ values were collected and sorted in ascending order to define vector **y**_*s*_. From **y**_*s*_, we defined new vectors

(A.4) $$\begin{array}{l} \text{z}=4.7\sin^{2}{\text{y}}_{s} \end{array}$$

and

(A.5) $$\begin{array}{l} {\text{y}}_{T_{2}}\left(n\right)=\frac{{\text{y}}_{T_{2}}\left(n-1\right)+\sqrt{{\left({\text{y}}_{s}^{2}\left(n\right)-{\text{y}}_{s}^{2}\left(n-1\right)\right)}^{2}+{\left({\text{z}}^{2}\left(n\right)-{\text{z}}^{2}\left(n-1\right)\right)}^{2}}}{\left(\text{max }{\text{y}}_{T_{2}}-\text{min }{\text{y}}_{T_{2}}\right)\left(\text{max }{\text{y}}_{s}-\text{min }{\text{y}}_{s}\right)}\\ -\text{min }{\text{y}}_{T_{2}}+\text{min }{\text{y}}_{s} \end{array}$$

where the *n* term reference the *n*th element of the corresponding vector, and

(A.6) $$\begin{array}{l} {\text{y}}_{T_{2}}\left(1\right)=\sqrt{{\text{y}}_{s}^{2}\left(1\right)+{\text{z}}^{2}\left(1\right)} \end{array}$$

This completes the transformation of the *y* dimension into a rotation angle expressed in radians.

Finally, the elements of **y**_*T*_2__ were returned to their original sort order and recombined into *frequency* × *orientation* coordinates.
